# Do Bioactive Food Compound with *Avena sativa* L., *Linum usitatissimum* L. and *Glycine max* L. Supplementation with *Moringa oleifera* Lam. Have a Role against Nutritional Disorders? An Overview of the In Vitro and In Vivo Evidence

**DOI:** 10.3390/nu13072294

**Published:** 2021-07-02

**Authors:** Rosângela dos Santos Ferreira, Lígia Aurélio Bezerra Maranhão Mendonça, Cristiane dos Santos, Priscila Aiko Hiane, Rosemary Matias, Octávio Luiz Franco, Ademir Kleber Morbeck de Oliveira, Valter Aragão do Nascimento, Arnildo Pott, Cristiano Marcelo Espinola Carvalho, Rita de Cássia Avellaneda Guimarães

**Affiliations:** 1 Graduate Program in Biotechnology, S-Inova Biotech, Catholic University Dom Bosco-UCDB, Campo Grande 79117-010, MS, Brazil; rosangela.ferreira@ufms.br (R.d.S.F.); lmendoncanutri@gmail.com (L.A.B.M.M.); cristinyba@gmail.com (C.d.S.); ocfranco@gmail.com (O.L.F.); cristiano@ucdb.br (C.M.E.C.); 2 Graduate Program in Health and Development in the Central-West Region of Brazil, Federal University of Mato Grosso do Sul-UFMS, Campo Grande 79079-900, MS, Brazil; priscila.hiane@ufms.br (P.A.H.); aragao60@hotmail.com (V.A.d.N.); 3 Graduate Program in Environment and Regional Development, University Anhanguera Uniderp, Campo Grande 79035-470, MS, Brazil; mdr@anhanguera.com (R.M.); akmorbeckoliveira@gmail.com (A.K.M.d.O.); 4 Graduate Program in Genomic Sciences and Biotechnology, Center of Proteomic and Biochemical Analysis, Catholic University of Brazilia, Brasília 70790-160, DF, Brazil; 5 Institute of Biosciences, Federal University of Mato Grosso do Sul-UFMS, Campo Grande 79079-900, MS, Brazil; arnildo.pott@gmail.com

**Keywords:** oat, flaxseed, soya, *M. oleifera*, nutraceuticals, non-transmissible chronical diseases, intestinal inflammatory diseases, malnutrition, intestinal microbiota

## Abstract

Functional clinical nutrition is an integrative science; it uses dietary strategies, functional foods and medicinal plants, as well as combinations thereof. Both functional foods and medicinal plants, whether associated or not, form nutraceuticals, which can bring benefits to health, in addition to being included in the prevention and treatment of diseases. Some functional food effects from *Avena sativa* L. (oats), *Linum usitatissimum* L. (brown flaxseed), *Glycine max* L. (soya) and *Moringa oleifera* have been proposed for nutritional disorders through in vitro and in vivo tests. A formulation called a bioactive food compound (BFC) showed efficiency in the association of oats, flaxseed and soy for dyslipidemia and obesity. In this review, we discuss the effects of BFC in other nutritional disorders, as well as the beneficial effects of *M. oleifera* in obesity, cardiovascular disease, diabetes mellitus type 2, metabolic syndrome, intestinal inflammatory diseases/colorectal carcinogenesis and malnutrition. In addition, we hypothesized that a BFC enriched with *M. oleifera* could present a synergistic effect and play a potential benefit in nutritional disorders. The traditional consumption of *M. oleifera* preparations can allow associations with other formulations, such as BFC. These nutraceutical formulations can be easily accepted and can be used in sweet preparations (fruit and/or vegetable juices, fruit and/or vegetable vitamins, porridges, yogurt, cream, mousses or fruit salads, cakes and cookies) or savory (vegetable purees, soups, broths and various sauces), cooked or not. These formulations can be low-cost and easy-to-use. The association of bioactive food substances in dietary formulations can facilitate adherence to consumption and, thus, contribute to the planning of future nutritional interventions for the prevention and adjuvant treatment of the clinical conditions presented in this study. This can be extended to the general population. However, an investigation through clinical studies is needed to prove applicability in humans.

## 1. Introduction

The growing escalation of non-transmissible chronic diseases (NTCD), intestinal inflammatory diseases (IID) and malnutrition has raised much concern in the general public and in public agencies related to nutrition and health. Taking into account that people affected by such diseases need a better quality of life, dietary intervention based on functional foods and nutraceutical medicinal plants are very important [1,2,3]. 

In this context, functional clinical nutrition arises, which is an integrative science, meaning it seeks aiming to understand the communication among all biological systems and to act in the prevention or treatment of organic imbalances. The clinical practice takes into account genetic and biochemical individuality, whereby functional foods, as well as medicinal plants and their combination, are utilized as dietary strategies [2,4,5,6,7,8].

Both functional foods and medicinal plants, whether associated or not, form nutraceuticals, which can bring benefits to health, besides being included in the prevention and treatment of diseases. Nutraceuticals contain isolated components, such as dietary fibers, poly-unsaturated fatty acids (PUFAs), proteins, peptides, amino acids, vitamins and antioxidant minerals, as well as dietary supplements consumed in different forms [1,9,10,11]. 

Nutraceuticals based on functional foods exert favorable effects, combining substances with bioactive activities, besides influencing health maintenance and correcting metabolic disorders, reducing the risk of diseases [12,13,14]. Nutraceuticals derived from medicinal plants utilize the pharmacological basis of botanical species, also with therapeutical coadjuvant purposes, and can be combined with functional foods, as part of a single nutraceutical formulation [15].

**Figure 1:**
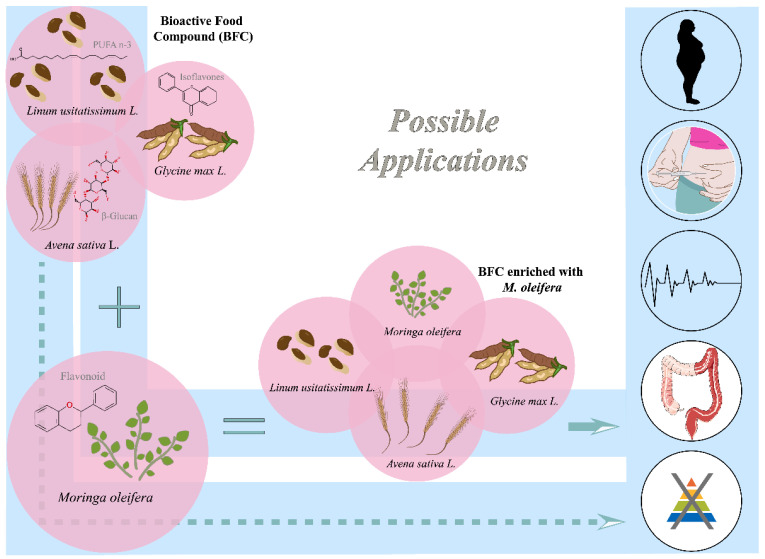
The nutritional and phytochemical composition of a bioactive food compound (BFC), *M. oleifera* and a BFC enriched with *M. oleifera* in NTCDs, IID and malnutrition.

Non-Transmissible Chronic Diseases (NTCDs) (obesity, cardiovascular disease and diabetes mellitus type 2 (DM2)), metabolic syndrome (MS), IID (intestinal inflammatory diseases)/colorectal carcinogenesis (CRC) and malnutrition have a close relationship with the metabolic–nutritional state (Figure 1).

Some effects from functional foods, such as *Avena sativa* L. (oats), *Linum usitatissimum* L. (brown flaxseed), *Glycine max* L. (soya) and *Moringa oleifera,* have been presented for nutritional disorders through in vitro and in vivo tests [16,17,18,19,20], NCT04314258 (Table 1 and Table 2). In the literature, there is vast knowledge of the benefits of these functional foods. However, a formulation, called a bioactive food compound (BFC), showed efficiency in the association of oats, flaxseed and soy for dyslipidemia and obesity [17,18].

Therefore, our research group intends to discuss the effects of BFC in other nutritional disorders as well as the beneficial effects of *M. oleifera* in the previously mentioned clinical conditions. In addition, we hypothesized that BFC supplementation with *M. oleifera* could present a synergistic effect and play a potential benefit in nutritional disorders.

In this narrative review, we used eligibility criteria based on studies on “functional foods”, (oat, flaxseed, soya and *M. oleifera*), “obesity”, “cardiovascular disease”, “diabetes mellitus type 2”, “intestinal inflammatory diseases”, “colorectal carcinogenesis” and “malnutrition”, associated with each other by the Boolean descriptor “AND” and selected from the Health Sciences Descriptors. A bibliographic search was used for ClinicalTrials.gov, Cochrane, Europe PMC, the MEDLINE^®^/PubMED^®^ database, MDPI, Scielo, Science Direct by Elsevier, Wiley online library, Springer–Nature database, Taylor & Francis, BMC and Hindawi, conducted, preferably, in 2010 until 2021.

Finally, this review raises the possible applicability of a BFC enriched or not with *M. oleifera* to act in important nutritional disorders that affect the world population.

## 2. Current Status of Knowledge

### 2.1. Bioactive Food Compound (BFC) and M. oleifera Nutraceuticals

A BFC is a food formulation prepared from functional foods, such as oats (*Avena sativa* L.), brown flaxseed (*Linum usitatissimum* L.) and soya (*Glycine max* L.) (Table 1). A patent application has been made for this BFC, which was triturated and homogenized at a ratio of 2:1:1 [19].

BFC have high nutritional quality, are rich in proteins and PUFAs and have an adequate n-6/n-3 ratio and dietary fibers, such as soluble fibers, which include lignan and β-glucan [19]. As they presents high versatility, BFC have been used in individuals with metabolic alteration, demonstrating effective control of the levels of triglycerides and LDL-cholesterol (low-density lipoproteins) [18]. The hypolipidemic character of BFC was shown by low atherogenicity and thrombogenicity as well as an adequate ratio between hypo- and hypercholesterolemia, PUFA: SFA and ω6: ω3, thus, demonstrating a high quality index of the BFC lipid fraction [19]. These results demonstrated the effects of fatty acids on the cholesterol metabolism and a low risk of developing cardiovascular diseases. BFC showed a high nutritional content because it has a high protein (24.27%) and fiber (7.98%) content, with a total energy value (kcal.100 g^−1^) of 335.25 [19]. In addition, this food formulation presented positive effects on the reduction of anthropometric parameters [17].

### 2.2. Moringa oleifera Lamarck

*M. oleifera* (syn. *M*. *ptereygosperma* Gaertn.) is a medicinal plant of the family *Moringaceae*, it is native to the sub-Himalayan region in the north of India, Pakistan, Africa, Asia Minor and Arabia [52,53] and has been introduced in other parts of the world [54]. It is a food plant with multiple medicinal uses. Medicinal uses of different parts of the plant, including leaves, roots, stem bark, flowers, pods and seeds, have been reported. Ethnobotanical studies confirmed *M. oleifera*’s anti-inflammatory, antihypertensive, diuretic, antimicrobial, antioxidant, antidiabetic, antihyperlipidemic, antineoplastic, antipyretic, antiulcer, cardioprotective and hepatoprotective properties. In vitro and in vivo studies reinforced these pharmacological properties through the action of the secondary metabolites, including antioxidant compounds, such as ascorbic acid, flavonoids, phenolics and carotenoids, present in the plant [55,56].

In addition, *M. oleifera* has a significant nutritional composition that is high in protein (about 19–29%), dietary fiber (about 19–37%) with about 205–350 cal/g of energy [57,58]. In addition micronutrients, such as iron, magnesium and folate, as well as vitamins of the B complex, such as B6, and vitamins A, C and E [57]. The leaves of this plant, as well as the pods and seeds, have a variety of essential phytochemicals that provide this nutritional characteristic, such as vitamins A, B, C, D and E, in addition to folic acid, pyridoxine and nicotinic acid [55,57].

In summary, the functional effects of BFC and *M. oleifera* may represent potential action in nutraceutical strategies for the nutritional management of NCDs, IID/CRC [59,60,61] and malnutrition [62] Figure 1.

### 2.3. Effect of Bioactive Food Compound (BFC) and M. oleifera on Non-Transmissible Chronic Diseases (NTCDs)/Metabolic Syndrome (MS)

Among the NTCDs, obesity is a nutritional disorder, arising from the imbalance between food intake and energy output, causing excessive body fat. Studies revealed that the effects of the increased liberation of free fatty acids, inflammatory cytokines (interleukin 6 and TNF-α (tumoral necrosis factor alfa)), NF-κB (nuclear factor kappa B) and other byproducts of adipocytes, generated by the excess of adiposity, are due to the concentration of abdominal or central fat [63]. Obesity, now considered a worldwide crisis, is strongly associated with the risk of MS, DM2, cardiovascular diseases and cancer [64].

Therefore, dietary resources are necessary to help in the treatment of the effects and consequences of obesity. Nutritional recommendations are beneficial, such as the daily intake of soluble fibers, especially β-glucan, found in high concentrations in oat bran. The intake of fibers provides a convenient solution for the risk coming from obesity, as their solubility interferes with satiety. In addition, the constitution of β-glucan includes molecules of glucose with the bonds β-(1-3) and β-(1-4), which establish physicochemical properties that can act in the reduction of systemic arterial pressure, serum glucose, total cholesterol and fractions and triglycerides as well as increase the fraction of High-Density Lipoprotein (HDL-c) [65,66,67,68,69,70].

It is believed that the hypolipemiant effect of oats is related to the high viscosity, which can interfere with the uptake of biliary acids, lowering the rate of intestinal cholesterol uptake [71,72], thus, reducing the quantity of chylomicrons and, as a consequence, reducing the circulating cholesterol [73]. Furthermore, the effects of normalization of the lipidic profile of soluble fibers, such as β-glucan, are associated with their capacity to raise the level of fermentation in the colon, producing short-chain fatty acids (SCFA) [74], which indirectly reduce blood cholesterol by inhibiting the hepatic synthesis of cholesterol [75].

Oats also act on lipidic alterations arising from the elevation of insulin, as reported by El Rabey, Al-Seeni and Amer (2013) [76] and Aleixandre and Miguel (2016) [77]. In this case, there was a lower secretion of post-prandial insulin, which can result in reduced lipogenesis [78]. Insulin is responsible for the activation of the enzyme HMG-CoA reductase (3-hydroxy-3-methyl-glutaric-CoA reductase or HMGR), whose reduction can involve less synthesis of cholesterol [75] and a lower level of circulating triglycerides [73].

Another deleterious consequence of obesity is the alteration in the glycemic profile, characterizing a chronic state of hyperglycemia and a weak sensitivity to insulin. The usage of oats contributes to the maintenance of the seric glucose levels, majorly attributed to its high content of soluble fibers [79,80,81]. The fiber β-glucan is the one mainly responsible for anti-hyperglycemic and hypoglycemic effects. This feature is due to the capacity of β-glucan to enhance intestinal viscosity and diminish glucose uptake [80,81]. Furthermore, the anti-hyperglycemic and hypoglycemic properties of this soluble fiber are also attributed to its high content of chromium (Cr)—a mineral that acts as an insulin enhancer and, consequently, improves blood glucose levels [82].

The intake of flaxseed can also repair damage in the lipidic and glycemic profile, as it is a functional food with a high PUFA content [83]. PUFAs can contribute to reducing specific inflammatory markers and cytokines, ensuring a general improvement of the endothelial function and, consequent, cardioprotective [84,85] and anti-hyperglycemic effects [86,87,88].

One of the mechanisms that can explain this function is the catabolic capacity promoted by eicosapentaenoic acid (EPA) and docosahexaenoic acid (DHA) that culminates in the reduction of the lipoproteins containing the apolipoprotein B-100 (ApoB). In addition, both suppress the production of hepatic ApoB, stimulate the plasmatic depuration of triglycerides via lipoprotein lipase (LPL), raise Very Low-Density Lipoprotein (VLDL-c), reduce the synthesis of LDL-cholesterol and attenuate postprandial lipemia [89].

The lipidic characteristic of flaxseed, which is one of the best food sources of essential FAs, with a richness of phenolic and antioxidant compounds [90,91], makes this functional food an excellent daily dietary alternative. According to Kuang (2020) [92], the effect of biscuits with flaxseed meal supplement, consumed at approximately 100 g per day, is sufficient to obtain the metabolic benefits on body weight, BMI and TG for overweight and obese individuals.

Flaxseed has a high content of soluble fibers, above all lignan, which contributes even more to the normalization of the lipidic profile. The effect is attributed to the capacity of this fiber to modulate 7-α-hydroxylase and acyl-CoA cholesterol transferase, involved in the regulation of liver cholesterol and its conversion into biliary acids, contributing to a higher depletion of cholesterol [75].

The soluble fibers of flaxseed also allow intestinal glucose uptake to be delayed, which can attenuate the need for insulin production and, as a consequence, diminish its synthesis. Another mechanism involved in the normalization of the glycemic profile is related to the fact that lignan suppresses the gene expression of phosphoenolpyruvate carboxykinase (PEPCK), which is related to the production of glucose, through gluconeogenesis, helping in glycemic control. The effects of regularization of the glycemic profile have also been attributed to PUFAs found in brown flaxseed [88].

Considering the lipidic and glycemic consequences of obesity, soya also exerts beneficial effects. These effects are mainly attributed to the rich content of isoflavone, fibers, oligosaccharides, phytosterols, lectins and phytic acid in this legume [93,94,95,96,97]. The effect on weight control is more pronounced due to isoflavone, which has chemical structures similar to endogenous estrogens, resulting in the inhibition of lipogenesis and adipogenesis [96,98].

Studies also showed that soya contains bioactive peptides, which exert hypolipidemic, anti-hypertensive, antioxidant and anti-inflammatory activities, thus demonstrating their broad physiological function [99,100]. For example, the hypocholesterolemic peptide can act as a competitive inhibitor of the major rate-limiting enzyme in cholesterol biosynthesis—3-hydroxy-3-methylglutaryl CoA reductase (HMGR). It can also increase LDL uptake in the sterol regulatory element-binding protein 2 (SREBP2) pathway [99]. The excellent applications and responses of soya on the normalization of the lipidic profile [101] have led to its approval through a health claim proving its use in reducing the risk of NTCDs [102] and, moreover, CVD [99], as can be explained by the reduction of cholesterol due the formation of insoluble complexes by soy saponins. The insoluble complexes can form mixed micelles that inhibit the resorption of bile acids in the terminal ileum [103].

The functional effects of soya are also extrapolated to the nutritional management of the glycemic profile, since soya can act in increasing sensitivity to insulin and its production by beta-pancreatic cells, in addition to inhibiting intestinal glucose uptake [93]. In this case, specifically, isoflavone has the capacity to reduce the loss of beta cells of the pancreatic islets and also presents a high antioxidant effect. This effect, especially from genistein, inhibits tyrosine kinase (PTK), the protein involved in potential alterations in insulin secretion. The isoflavones also appear to regulate postprandial glycemia by blocking the activity of the enzymes α-amylase and α-glycosidase, in vitro [102].

This takes advantage of the effects of oats, flaxseed and soya on the normalization of the lipidic and glycemic profile, through their chemical and nutritional composition, providing strategies for the nutritional management and prevention of obesity, DM2, dyslipidemia/CVD and MS [61,104,105,106,107]. These functional foods and their different bioactive properties related to NTCDs can be associated with the nutrients present in *M. oleifera*, which presents excellent nutritional potential and broad pharmacological properties [20,31,108,109]

The chemical components present in *M. oleifera* are fundamental for health maintenance or improvement. The plant has been applied in lipid profile adjustment [110] in an animal model and demonstrated a reduction in the total cholesterol, triglycerides and LDL-c; hence, it can be used as an effective alternative in cases of dyslipidemia [111]. The hypolipidemic effect of *M. oleifera* can be attributed to the lower biosynthesis of cholesterol through the inhibition of HMG Co-A reductase (3-hydroxy-3-methyl-glutaric-CoA reductase or HMGR), the enzyme that regulates the levels of serum and tissue cholesterol. The capacity of *M. oleifera* to improve the lipid profile can be justified due to its high content of phenolic compounds, especially flavonoids, like rutin, quercetin and kaempferol, which also contribute to reducing the intestinal absorption of cholesterol [112].

In addition, *M. oleifera* also has β-sitosterol, a phytosterol with a cholesterol-like structure, except for the substitution of an additional C-24 alkyl group and/or a C-22 double bond. Studies have demonstrated that β-sitosterol can lower serum cholesterol levels by reabsorbing endogenous cholesterol, increasing its excretion in feces as neutral steroids [113]. Other beneficial effects of phytosterols include anti-inflammatory and antipyretic properties [114,115].

The functional effects of *M. oleifera* extend to the strategies for the nutritional management of DM2, taking advantage of its anti-hyperglycemic properties [116,117] as well as its antioxidant potential [118,119]. Therefore, *M. oleifera* is widely used in human and animal studies, whose primary scope is to determine the anti-hyperglycemic effects of the plant and at the same time its antioxidant property through evaluation of the activity of enzymes, such as SOD (superoxide dismutase), CAT (catalase) and GSH (glutathione peroxidase) [48,88,116,117,120]

The anti-hyperglycemic effect of *M. oleifera* can be attributed to its capacity to enhance the action of insulin [50,117]. This is because the plant has considerable quantities of bioactive phytochemicals (quercetin, kaempferol, chlorogenic acid and alkaloids), which act in synergy, increasing the secretion of insulin and leading to a better use of glucose by the tissues through blockage of hepatic gluconeogenesis [50].

Considering the pronounced bioactive effects of these functional foods and *M. oleifera*, as well as the risk factors related to visceral fat, hypertension, low levels of HDL-c and high levels of triglycerides and glucose, configuring MS [121,122,123], two nutraceutical options, BFC and *M. oleifera*, can be recommendable.

### 2.4. Alternative Therapies and/or Prophylactics for Intestinal Inflammatory Diseases (IID): Bioactive Food Compound (BFC) and M. oleifera

In addition to NTCDs, IIDs are also characterized by inflammatory processes. The particularity of these intestinal diseases involve prolonged inflammation of the digestive tract [124]. In this case, the nutraceutical strategies of BFC and *M. oleifera* can also intervene in the prevention and or nutritional treatment of celiac disease, ulcerative colitis, Chron’s disease, irritable bowel syndrome and even cancer, specifically CRC, one of the most worrying IIDs [60]. However, clinical trials are needed to prove this hypothesis.

The long-term dietary ingestion of oats or oat bran can confer beneficial outcomes on IIDs [125,126,127]. In celiac disease, for example, oats can be considered an excellent therapeutic nutritional indication, as they are nutritive and safe for a gluten-free diet [128]. However, oats should be chosen with caution, since contamination from other sources of cereals is the main problem faced by celiac patients [129].

The primary mechanism of action justifying the use of oats in the nutritional management of IIDs, including celiac disease, is regulating the bowel transit time and increasing the production of butyrate and/or other SCFAs by the intestinal microbiota, ensuring improved inflammatory and oxidant processes [129].

Considering that IIDs have, as their main pathologic characteristic, a severe inflammatory context, the prolonged consumption of brown flaxseed determines the increase of PUFAs, such as n-3, which is associated with a lower incidence of ulcerative colitis [25,130]. That is because PUFAs inhibit the synthesis of prostaglandins (PG) and leukotrienes (LT) via arachidonic FA. They also inhibit angiogenesis and adaptive immunological responses [131].

Furthermore, taking advantage of the antioxidant properties of flaxseed, there is a reduction in the severity of ulcerative colitis, with a reduction in goblet cell depletion, inflammation and scavenging ROS (reactive oxygen species) [39,132]. The reduction of the inflammatory process can be justified by the capacity of brown flaxseed to lower the TNF-α levels. The minimization of the oxidative process can be related to the fact that this seed acts in the inhibition of nitric oxide, through iNOS (nitric oxide inducible synthase), with negative regulation of IFN-γ (interferon-gamma) and TNF-α and an increase of interleukin 17 (IL-17) [39].

Considering the reduction of inflammation and oxidative stress, both highly present in IIDs, studies revealed that the isoflavones found in soya (in the form of aglycone, which is bioavailable and rich in daidzein) have an effect on intestinal immunity, since they diminish the inflammation and colon tissue damage, thus, avoiding colitis [133,134]. In addition, the moderate ingestion of isoflavones in the diet can be beneficial in remission cases of ulcerative colitis, as shown in an animal model [135].

The anti-inflammatory, immunomodulator and antioxidant properties of *M. oleifera* are also attributed to the content of phenolic compounds, especially bisphenols and flavonoids, whose positive effects on IIDs were reported in an animal model [136]. However, there is a clinical trial evaluating the effects of *M. oleifera* leaves on the blood antioxidant status. Lipid profiles and the blood glucose level will be evaluated (NCT04314258). The anti-inflammatory/antioxidant effect of *M. oleifera* is mediated by the transcription factor Nrf2 (nuclear factor erythroid 2–related factor 2). In addition, the plant can lower nitric acid, through iNOS, TNF-α, myeloperoxidase (MPO) and proinflammatory interleukin, such as interleukin IL-6, all markers of inflammatory/oxidative processes [137].

The contribution of the functional foods composing BFC and *M. oleifera* can occur by means of chemopreventive and or chemotherapeutic properties, with anticarcinogenic and antiangiogenic effects [59]. Thus, the use of BFC and *M. oleifera* in the nutritional management of CRC can be justifiable due to the high content of soluble fibers, lignan and β-glucan present in flaxseed and oats [59,81,138]. Clinical trials are encouraged to confirm this hypothesis. These soluble fibers have high prebiotic potential, modifying the composition of the intestinal microbiota [139] and, when undergoing a fermentative process by anaerobic bacteria in the colon, they ensure the synthesis of SCFA [139,140]. These FAs can contribute to lowering the pH of the intestinal lumen for the elimination of toxins and proliferation of colon epithelial cells [139] and enhance an effect of prevention and reduction of a possible inflammatory process that would lead to the development of lesions [59].

In turn, soya is used in the prevention and nutritional treatment of CRC, justified by its high content of isoflavone, specifically genistein, daidzein and enterolactone. These substances can inhibit angiogenesis, diminish cell proliferation and increase the apoptosis of cancer cells [27,28,141].

*M. oleifera* also has antitumoral/antiproliferative properties, since this species can act on the induction of apoptosis of tumoral cells. The mechanism of action of *M. oleifera* appears to be related to blocking the progression of the tumoral G2/M cycle. That effect can be attributed to the plant-rich chemical composition with various anti-cancer compounds, namely eugenol, isopropyl isothiocynate, D-allose and hexadeconoic acid ethyl ester, with long chain hydrocarbons, a sugar moiety and an aromatic ring [47]. Other antitumoral/antiproliferative mechanisms of *M. oleifera* include the capacity to increase oxidative stress and produce an alteration in the cell tumoral cycle, due to apoptosis [142].

### 2.5. Nutraceuticals for Malnutrition: Bioactive Food Compound (BFC) and M. oleifera

The investigation and understanding of the effects presented by the food formulation BFC and *M. oleifera* can also direct their application in situations where malnutrition is evident in hypercatabolic diseases, which leads to alterations of body composition and physiological functions caused by clinical conditions, which can cause a loss of appetite or hinder food ingestion [57,62].

To intervene in an adjuvant way in both prevention and treatment of metabolic alterations that can lead to malnutrition, oat and its products stand out, given that numerous clinical trials demonstrate that foods and supplements with soluble fibers, particularly the β-glucan fraction, are well accepted and widely consumed [118,143,144].

Flaxseed is another functional food that is important in the nutritional management of malnutrition, as it is a source of high-quality functional protein in addition to containing lignans, gum and phenolic compounds. The protein of flaxseed can be used by patients with malnutrition associated with cancer, burns, liver failure and chronic and acute diarrhea. The components of flaxseed can be developed into various fortified functional products, owing to a high impact in the protection and treatment of various chronic diseases [145].

Foods supplemented with soya are used as dietetic formulations for the treatment of undernourished people [29,30,146]. That is because soya has high nutritional potential, as it is rich in proteins, carbohydrates and lipids plus minerals, such as potassium, and vitamins, such as riboflavin, choline, thiamin and pantothenic acid [147]. Soya has been incorporated in mixtures to prevent malnutrition and to improve the nutritional state of children [148]. The nutritional composition of BFC demonstrated high protein and energetic content and can be recommended for the condition of malnutrition [19].

*M. oleifera* is a nutraceutical alternative and a particularly relevant resource against malnutrition. That is because *M. oleifera* is rich in macro and micronutrients [57,149,150,151]. The plant is also considered to be a moderately adequate source of calcium, niacin (B3), protein, essential amino acids (threonine, valine, methionine, leucine, isoleucine, phenylalanine, histidine, lysine and arginine) and dietary fiber [57,108,149,152].

Due to its expressive nutritional potential, *M. oleifera* has been used in dietary formulations for the enrichment of everyday food, aiming at replacing nutrients in cases of malnutrition and anemia [149,153]. Given the phytochemical composition and nutritional value of *M. oleifera* demonstrated in vitro and in vivo, the use of this medicinal plant can positively impact the nutritional status in humans [16,154]. A clinical study showed the efficiency of *M. oleifera* leaf powder in improving the nutritional status of people living with human immunodeficiency virus (PLHIV) [155]. Another study showed the supplementation of *M. oleifera* leaf powder, administered in children with malnutrition, demonstrating the effectiveness in improving nutritional recovery [151]. On the other hand, the consumption of 14 g of *M. oleifera* did not improve the nutritional status and body composition of malnourished individuals [108,155,156,157]. Therefore, further clinical studies are needed to indicate *M. oleifera* for malnutrition.

Hence, the impact of incorporation or supplementation of oats, brown flaxseed, soya and *M. oleifera* is a critical dietary strategy in controlling malnutrition.

## 3. Functional Dietary Modulation of the Intestinal Microbiota from the Nutraceuticals Bioactive Food Compound (BFC) and *M. oleifera*

The intestinal microbiota is an essential component in the human body ecosystem and possibly a health modulator. The intestinal and general homeostasis of the organism is directly linked to the integrity of the intestinal mucosal barrier, thus, avoiding bacterial translocation. This can impede tissue lesions, infection by pathogens and the development of diseases [158].

Various studies revealed that functional alterations and changes in the composition of the gut microbiome are associated with NTCDs and IID, such as obesity [159,160], diabetes mellitus [161,162,163,164], cardiometabolic disease [165], irritable bowel syndrome [166,167,168], CRC [169,170] and malnutrition [171].

Thus, it has been pointed out that, for modulation of the microbiota, it is necessary to consider geographic, environmental, genetic and dietetic factors, in addition to the standardization of the analytical procedures. For functional characterization, it is also necessary to update the catalogues of human intestinal micro-organisms [172,173].

Regarding dietary factors, food habits can be improved by adding nutraceuticals containing soluble fibers [174], PUFAs [175], isoflavones [43] and medicinal plants. These induce functional changes and alter the composition of the intestinal microbiota [176], thus, highlighting the possibility of using nutraceutical BFC and *M. oleifera*.

Global dietary recommendations emphasize the intake of oats, due to the physicochemical properties and physiological responses in promoting health. The efficiency of the grain is best when associated with a hypolipidic diet, with a low content of saturated fatty acids, thus, contributing to reducing the risk of NTCDs [174]. The consumption of oats contributes to the health of the bacterial community of the distal colon, by increasing the viscosity in the gastrointestinal tract, through β-glucan and resistant starch, one of the main determinants of favorable metabolic effects [177]. There is evidence suggesting that oats raise the production of SCFA in the large intestine, triggering the homeostasis of its microbiota [65,178].

The soluble fiber of oats influences an increase in the relative abundance of potentially beneficial bacteria, such as bifidobacteria and lactobacilli. It, thus, contributes in the modulation of the ecological processes that regulate the structure and function of the community of intestinal microbiome toward a profile of health promotion [179,180].

The modulation of the intestinal microbiota can also occur by using flaxseed, which is rich in multiple bioactive compounds, especially lignans [181,182], PUFAs and secoisolariciresinol diglucoside (SDG), with anti-inflammatory properties [183]. Lignans, which are non-steroidal phytoestrogens, are metabolized by intestinal bacteria to access the systemic circulation in humans [184]. The antioxidant and anti-inflammatory power of flaxseed is provided by SDG, especially when ground.

SDG liberates secoisolariciresinol (SECO), which produces dihydroxyalododiol by demethylation by the microbiota and, by dihydroxylation, results in enterodiol and dehydrogenation produces enterolactone [182,185,186]. All these metabolic processes occur by the bio transforming action of intestinal bacteria, such as *Ruminococcus bromii* and *R. lactaris* [187,188], *Lactobacillus casei* and *L. acidophilus*, acting for the digestion of the whole flaxseed, to increase the bioaccessibility of the enterodiol [189]. The supplementation of flaxseed in the diet can alter the colon bacteria, with a significant increase of *Prevotella* spp. and decrease of *Akkermansia muciniphila* [42]. Further studies are necessary to better understand the mechanisms of action of dietary flaxseed in the intestinal microbiome in healthy or ill people.

Soya also has effects on the population and composition of the intestinal microbiota. The components of soya, such as isoflavones (daidzein, genistein and glycitein), can increase levels of bifidobacteria and lactobacilli and alter the relationship between Firmicutes and Bacteroidetes, thereby, diminishing the risk of diseases, leading to beneficial effects on human health [43]. Foods based on soya can serve as sources of nutrients and energy that support the growth and maintenance of intestinal bacteria [190,191]. A study on the supplementation of 20% of soy protein showed an alteration in the bacterial composition of Firmicutes, with the increase and decrease in abundance of *Enterococcus* and of the levels of *Ruminococcus* and *Lactobacillus*, respectively [192].

The oligosaccharides and fibers of soya have prebiotic properties and can reach the colon intact without being digested [193,194]. Soya fibers are the main byproduct of the fermentation of non-starch polysaccharides by the anaerobic microbiota in the intestine; they alter the level of SCFAs, such as butyrate [195,196]. There is evidence that the oligosaccharides and fibers can contribute to favorable effects on the intestinal microbiota [43]. They also help to increase *Bacteroides*, *Flavonifractor*, *Barnesiella*, *Oscillibactor* and *Alistipes* and cause a significative drop in the abundance of *Ruminococcus*, *Lactococcus*, *Akkermansia*, *Hydrogenoanaerobacterium* and *Parabacteroides* [197].

*M. oleifera* contributes to restoring the number of lactobacilli and bifidobacteria in the cecal portion, thereby, modulating the intestinal microbiota. The phenolic compounds demonstrated promotion of the growth of probiotics, such as *Lactobacillus*, and interfering with the growth inhibition of pathogenic bacteria, such as *Escherichia coli* [51].

**Figure 2:**
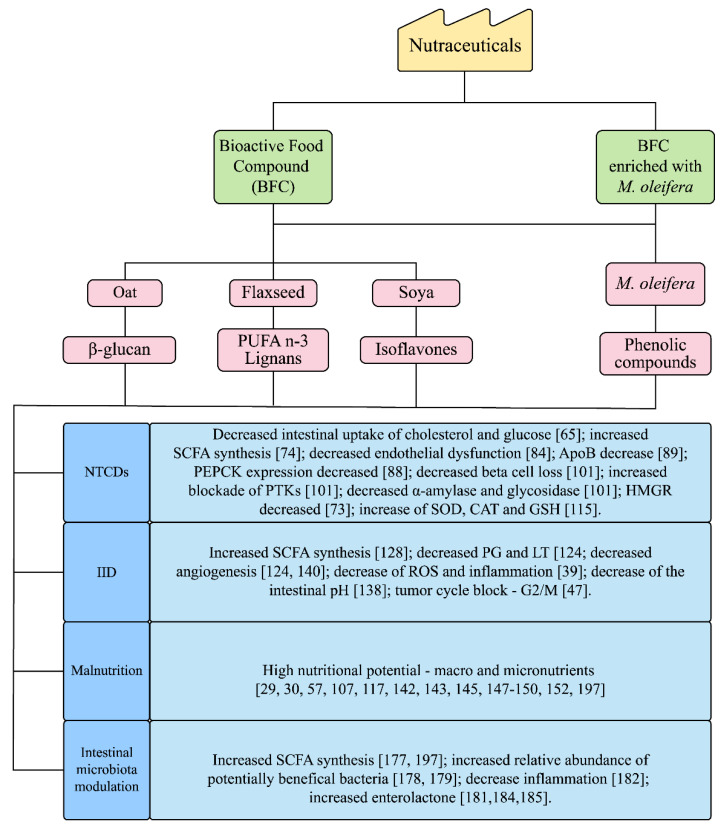
BFC and *M. oleifera* physiological and metabolic effects proposed. Short-chain fatty acids (SCFA); Apolipoprotein B (ApoB); Phosphoenolpyruvate carboxykinase (PEPCK); Tyrosine kinase (PTKs); 3- hydroxy-3-methyl-glutaric-CoA reductase (HMGR); Superoxide dismutase (SOD); Catalase (CAT); Glutathione peroxidase (GSH); Prostaglandins (PG); Leukotrienes (LT); and Reactive oxygen species (ROS).

Finally, there is a wide clinical indication for functional foods (oats, flaxseed and soy) and *M. oleifera* in nutritional disorders. Therefore, nutritional interventions, such as BFC and *M. oleifera,* may constitute viable alternatives in the management of chronic non-communicable diseases, inflammatory bowel diseases, malnutrition and modulation of the intestinal microbiota (Figure 2).

## 4. Concluding Remarks

BFC contains soluble fibers, polyunsaturated fatty acids, isoflavones, antioxidants, has a high nutritional value and has an impact on reducing obesity indicators in addition to adjusting the lipid profile. The BFC composition may also be signaling immunomodulatory effects on the intestinal microbiota, with preventive and therapeutic action for colorectal cancer. Based on the in vivo and in vitro results, the effects described above can be attributed to *M. oleifera,* as well as in blood glucose control, as it is considered an antidiabetogenic agent.

Considering the traditional consumption of preparations with *M. oleifera*, it is possible to favor their association with other formulations, such as BFC. Moreover, these nutraceuticals can be easily accepted and can be used in sweet preparations (fruit and/or vegetable juices, fruit and/or vegetable vitamins, porridges, yogurt, cream, mousses or fruit salads, cakes and cookies) or savory (vegetable purees, soups, broths and various sauces), cooked or not. These formulations can be low-cost and easy-to-use. However, it is suggested that the association of bioactive food substances in dietary formulations can facilitate adherence to consumption and, thus, contribute to the planning of future nutritional interventions for the prevention and adjuvant treatment of the clinical conditions presented in this study and can be extended to the general population.

Therefore, our review study showed the possible applicability of BFC and *M. oleifera* in nutritional disorders. Evidence suggests that the high nutritional values and the presence of phytochemicals in *M. oleifera* leaves can potentialize the beneficial effects of BFC. Thus, we propose BFC enriched with *M. oleifera* in the management of chronic non-communicable diseases, inflammatory bowel diseases, malnutrition and modulation of the intestinal microbiota. However, an investigation through clinical studies is needed to prove its applicability to BFC as well as BFC enriched with *M. oleifera* for these nutritional disorders.

## 5. Patents

Patent application: Instituto Nacional da Propriedade Industrial, Brazil (number BR 10 2013 018,002 5 on 6 June 2013). The BFC was launched by Ministério do Desenvolvimento e Comércio Exterior, Brazil, August 2015.

## Figures and Tables

**Table 1:** BFC and *M. oleifera* nutraceutical bioactivity in clinical trials.

Physiological and Metabolic Effects	Population/Treatment Time/Applied Dose	References
**Oat**		
β-glucans reduced mean LDL-C and TC levels	83 participants, 8 weeks, boxes containing 28 sachets, 3 g β-glucans	[21]
Reduced LDL and TC levels	28 RCTs, ≥3 g/day, food products, ≥2 weeks	[22]
Reduced glucose, TC, triglycerides and BMI and waist-hip ratio	30 participants with MS, 37.26 years/8 weeks/15 g/day fiber intake oat bran	[23]
Improved nutritional status	1406 children between 6 and 59 months of age with uncomplicated SAM/12 weeks/30 g oat-RUTF	[24]
**Flaxseed**		
GF and FO attenuated systemic inflammation	75 patients with UC, GF (30 g/day) and FO (10 g/day), 12 weeks	[25]
Patients with mild to moderately severe UC	18 years and over, phase 2, FLC, 300 mg FLC, 12 week	NCT02201758
Effective in amelioration of some symptoms of MS and decrease BP and lipid peroxidation	60 participants/aged 30 to 60 years/ 25 mL/d FO/7 week	[26]
**Soya**		
Add genistein FOLFOX or FOLFOX-Bevacizumab. Efficacy results are notable	13 participants metastatic colorectal cancer, 7 days, 2 weeks	[27]
Reduced risk for overall colorectal cancer	901 participants with colorectal cancer, 2669 participants as control, high intake of total soy products or dietary isoflavones	[28]
Added genistein to the regimens of FOLFOX or FOLFOX-Avastin	Participants with metastatic (stage IV) colorectal cancer, Phase 2	NCT01985763
Soybean fortified meal improved the nutritional status of the children malnutrition	1546 children aged 6–23 months/1 month of supplementation/3 meals per day with 40g CSB per meal	[29]
Nutrition in the management of HIV/AIDS. Higher total blood protein, blood white protein, level of blood hemoglobin and higher CD4 cell count	47 PLWHAs/Nutritional and intervention Education Program/100 g soybean/day	[30]
***Moringa oleifera***		
Decreased postprandial glucose response. *M. oleifera* leaf powder could be a hypoglycemic herbal drug	17 Saharawi diabetics and 10 healthy subjects, 20 g of *M. oleifera* leaf powder	[20]
*M. oleifera* infusion effect in hot water twice daily on the blood glucose plasma lipids level and blood anti-oxidant status. Comparison of the lipid profiles of both healthy and hyperglycemic participants	RCS, 18–65 years (adult, older adult), 103 participants—*M. oleifera* leaves infused	NCT04314258
Investigation of the effects of *M. oleifera* supplementation on the levels of inflammatory markers, specifically the hsCRP, hgbA1c level and clinical outcome in diabetic patients through a cohort study	56 participants, supplementation of *M. oleifera*	NCT02308683
Potential lowering effect on both SBP and DBP, postprandial follow-up	41 healthy participants, 120 g of cooked *M. oleifera* leaves	[31]
Glycemic control and no adverse effects in T2DM. Tendency on blood pressure reduction in T2DM	Therapy-naïve T2DM with the duration of diabetes of less than 5 years, 20–70 years, 8 g, day, 40 days of *M. oleifera* leaf capsules	[32]
Increased insulin secretion, potential agent in the treatment of type 2 diabetes	10 healthy subjects, 24–34 years, 4 g capsules *M. oleifera* leaf powder	[33]
Antipyretic effects	A CS, an 18-month-old girl, 40 mL of warm water extract of 5 g	[34]
Investigated weight gain and hemoglobin and vitamin A status	Adolescent girls, 150 gm of Sajna shak/bora (*Moringa*) 5 days/week/6 months	NCT04156321
Reduction in anemia cases	95 anemic children/200 g/m/*M. oleifera* leaf powder/6 months	[35]
*M. oleifera* improved parameters associated with obese-DM2	24 obese DM2/17 women and 7 men, 20–60y/22w *M. oleifera* leaves	[36]
To evaluate the in vivo bioavailability of key nutrients and bioactives and biological activities of the leaves, malnourishment prevention	10 participants/18 Years and older (Adult, Older Adult)/Healthy males or females/corn *Moringa* Diet	NCT04092517

BMI—Body Mass Index; BP – Blood Pressure; CD4—Cluster of Differentiation 4; CS—Case Study; CSB—corn-soy blends; FLC—Flaxseed Lignan-enriched Complex; FO—Flaxseed Oil; GF—Grounded Flaxseed; HIV/AIDS—Human Immunodeficiency Virus/Acquired Immunodeficiency Syndrome; LDL-C—Low Density Lipoproteins—Cholesterol; MS—Metabolic Syndrome; ML *M. oleifera* leaves; MS—*M. oleifera* seeds. PLWHAs—people living with HIV/AIDS; RCS—Randomized Clinical Study; RCT—Randomized Controlled Trials; SAM—Severe Acute Malnutrition; T2DM—Type 2 Diabetes Mellitus; TC—Total Cholesterol; UC—Ulcerative Colitis; High sensitivity C-reactive protein (hsCRP); systolic blood pressure (SBP); diastolic blood pressure (DBP); and HbA1c (Hemoglobin A1c).

**Table 2:** BFC and *M. oleifera* nutraceutical bioactivity: in vitro and animal model bioassays.

Physiological and Metabolic Effects	Population/Treatment Time/Applied Dose	References
**Oat**		
Hypolipidemic	Wistar–Lewis male rats—30 days/oat flake powders: dose of 5 g kg^−1^ body weight per day. β-glucan extracted and purified dose of 0.3 g kg^−1^ body weight per day	[37]
Improved values of antioxidative potential markers; positive effect on the colon tissue of healthy rats with LPS-induced enteritis	72 old male Sprague–Dawley rats: Male Sprague—Dawley rats/6 weeks/84.0% Low molecular weight oat Beta-glucan	[38]
**Flaxseed**		
Reducing goblet cell depletion, scavenging oxygen-derived free radicals, reduce neutrophil infiltration that may be attributed due to decreasing IFN-γ and TNF-α and increasing IL-17 levels	BALB/c mice induced colitis/6 days/150, 300 and 500 mg/kg/day	[39]
Antihyperglycemic effect mediated through inhibition of ROS	Male Wistar rats/21 days/200 and 400 mg/kg/EELU	[40]
Improved glucose utilization; increased glucose-6-phosphate dehydrogenase; reduction of PPHG	Male Swiss mice/21 days/2, 1 mg flaxseed powder	[41]
Alters the baseline colonic microenvironment of healthy mice, which may modify subsequent mucosal microbial defense and injury-repair responses leading to altered susceptibility to different gut-associated diseases	C57Bl/6 male mice/3 weeks/10% flaxseed	[42]
**Soya**		
Decreased body weight and the plasma TG and LDL concentrations. Decreased in activity of mTORC1. Suppressed lipogenesis and adipogenesis, potential mechanism of soy isoflavones regulating lipid homeostasis.	64 Male rats/4 weeks/Basal diets + 50 mg/kg; 150 mg/kg; 400 mg/kg doses of soy isoflavones	[43]
Reduced the body weight gain and related biomarkers. Fat deposits, dyslipidemia, hyperglycemia and fatty liver were ameliorated by dietary genistein.	Male C57BL/6J mice/(*n* = 15, 16 weeks) 0.25% genistein (Study 1) and (*n* = 75, 18 weeks) 0.2% and 0.067% (Study 2) dose-response effect of genistein	[44]
Altered the microbial composition and modulated the metabolic pathway of the microbial metabolism in the colon. Serum levels of IgG and IgM were significantly increased in FF group pigs (*p* < 0.05). FF significantly decreased the abundances of Bacteroides and Verrucomicrobia in the duodenum and decreased the abundances of Bacteroides, Proteobacteria and Verrucomicrobiain in the colon and significantly increased the abundances of Firmicutes and Actinobacteria (*p* < 0.05). Serum immunity and expression of genes related to gut immunity were associated with bacterial strains at the family level	48 growing barrow pigs/2 feeding groups (*n* = 24 each, UF and FF)	[45]
***Moringa oleifera***		
Antioxidant, hypolipidemic and antiatherosclerotic activities, (*p* < 0.05) lowered the cholesterol levels and reduced the atherosclerotic plaque formation to about 50% and 86%, respectively	Rabbit/12 weeks/*M. oleifera* leaf extract	[46]
Anti-cancer activity/MDA-MB-231 and HCT-8 cancer cell lines	In vitro/250 mg of extracts were dissolved in 1.0 mL of ethanol	[47]
Prevention of cognitive damage due to chronic hyperglycemia and oxidative stress	88 Wistar rats/14 days/2 e 4% de ML/MS.	[48]
*Moringa* leaf extract reversed hepatic insulin insensitivity, up-regulation of genes involved in insulin receptors and glucose uptake in the liver	10 hyperinsulinemic male rats/4 weeks/300 mg aqueous extract of *M. oleifera* leaves/kg body weight.	[49]
Reduction in blood glucose and HbA1c levels and an elevation in serum insulin and hepatic glycogen levels.	Wistar rats/60 days/70% *M. oleifera* leaf extract (100, 250 and 500 mg/kg b.wt./day)	[50]
Regulation of weight gain and inflammation associated with high-fat-induced-obesity through gut bacteria modulation.	45 Swiss albino mice/3 months/(200 mg/Kg *M. oleifera* leaf extract	[51]

BMI—Body Mass Index; EELU—Ethanolic Extract of Seeds of *Linum usitatissimum*; SAM—Severe Acute Malnutrition; oat-RUTF—oat-Ready-to-use therapeutic food; PPHG—postprandial hyperglycemia; CSB—corn-soy blends; PLWHAs—people living with HIV/AIDS; mTORC1—mechanistic target of rapamycin complex 1; MGO—Methylglyoxal; FF—fermented complete commercial soybean meal; UF—commercial soybean meal; ML *M. oleifera* leaves; and MS—*M. oleifera* seeds.
